# The impact of internal control systems on the intensity of innovation and organizational performance of public sector organizations in Vietnam: the moderating role of transformational leadership

**DOI:** 10.1016/j.heliyon.2022.e08954

**Published:** 2022-02-16

**Authors:** Tu Thanh Hoai, Bui Quang Hung, Nguyen Phong Nguyen

**Affiliations:** School of Accounting, University of Economics Ho Chi Minh City, Ho Chi Minh City, Viet Nam

**Keywords:** Intensity of innovation, Internal control systems, Organizational performance, Public sector organizations, Transformational leadership, Vietnam

## Abstract

Drawing on the resource-based view, new public management theory, and innovation systems theory, this study examines the relationships between internal control systems (ICSs) and organizational performance in Vietnamese public sector organizations (PSOs), with particular emphasis on the mediating role of intensity of innovation and the moderating role of transformational leadership. Data from 319 PSOs in Vietnam corroborated our hypothesis that ICSs boost the intensity of innovation, which has a beneficial effect on organizational performance. Additionally, data demonstrated that intensity of innovation fully mediated the relationships between ICSs and organizational performance, and that transformational leadership reinforced the mediating relationships. The findings have implications for our understanding of the function of leadership and ICSs in managing innovation and promoting performance in PSOs in emerging markets.

## 1 Introduction

The global wave of innovation has resulted in a variety of pressing demands on management control practices. In emerging markets, particularly in public sector organizations (PSOs), innovation is valued and encouraged (Arundel et al., 2015; de Vries et al., 2018). Even a small innovation in the public sector can result in significant outcomes or effects extending beyond the public sector (Edler and Yeow, 2016; Edquist and Zabala-Iturriagagoitia, 2012). However, PSOs are criticized for being bureaucratic, excessively stable, stagnant, and conservative (Parker and Bradley, 2000). Therefore, these organizations' innovation activities are slow, fragmented, and asynchronous (Fagerberg et al., 2005). The low level of innovation in PSOs (Fagerberg et al., 2005) may be politically mandated and reversible within a short period or as a result of leadership practice (Helms, 2016). In this situation, leadership may be one aspect that requires additional consideration, as its importance has been widely recognized in the public management literature (Fernandez, 2005; Van Slyke and Alexander, 2006; Van Wart, 2014). Additionally, neo-institutional sociology has criticized public sector managers and organizations as passive adaptors to change (Modell, 2001). Therefore, PSOs may require transformational leadership to foster innovation. This is partly due to the fact that PSOs demonstrate a higher level of transformational leadership than scholars typically expect, and it has not had a detrimental effect on the prevalence of the practice of transformational leadership behaviors (Wright and Pandey, 2010).

Moreover, the strength of an organization's internal control system (ICS) is a critical factor affecting innovation (Freeman and Engel, 2007). An ICS comprises control mechanisms, which refer to the regulation of activities to ensure they adhere to the policies and goals established (Li et al., 2006). These mechanisms are intended to assist managers in effectively measuring innovation, providing feedback, and facilitating the sharing of resources and information among various departments (Hunziker, 2017). Furthermore, it is claimed that an ICS is vital for innovation in PSOs (Jaskyte, 2012), and it is an essential institutional driver of PSOs' performance (Babatunde and Dandago, 2014). Therefore, it is worth mentioning the potential benefits of ICSs for promoting organizational innovation in the public sector.

However, there are two schools of thought regarding the effect of ICSs on innovation. One believes that ICSs promote innovation (e.g., Brown and Martinsson, 2019; Li et al., 2006; Li et al., 2011; Shen et al., 2020), while the other believes the opposite (e.g., Abernethy and Brownell, 1997; Castiaux, 2007; Chan et al., 2020; Li et al., 2019). To be more specific, internal control mechanisms can influence the outcomes of knowledge exploitation during the innovation process (Li et al., 2006), thereby increasing the effectiveness of innovation (Shen et al., 2020). Since effective internal control enables organizations to establish appropriate strategic objectives, clarify the responsibilities of individual positions, increase efficiency, and mitigate conflict of interest, organizations may have additional human, material, and financial resources to innovate (Shen et al., 2020). However, Castiaux (2007) claims that ICSs do not promote or even hinder innovation since they have resulted in adverse outcomes in the knowledge generation process of innovation, preventing radical innovation.

While there in an ongoing debate regarding the impact of ICSs on innovation in PSOs worldwide, our paper contributes to the ongoing debate on whether ICSs bring innovation benefits to PSOs. Our study establishes the limit on the extent to which ICSs can act as a driver of innovation in PSOs, and that transformational leadership plays a moderating role in this relationship. In doing so, we developed a model of moderation mediation to demonstrate how ICSs can increase the intensity of innovation and organizational performance of PSOs in an emerging market by leveraging the moderating role of transformational leadership.

While recent studies on the relationship between transformational leadership and innovation have been quite extensive in emerging markets (e.g., Campbell, 2018; Moynihan et al., 2013; Wright and Pandey, 2010), it is not known how ICSs influence innovation with the facilitating role of transformational leadership. Particularly in the public sector, where tradition, experience, and conservative institutional solutions heavily influence leaders' decisions, resistance to creativity and innovation is pervasive (Vigoda-Gadot et al., 2005). Furthermore, public sector leaders are fearful of innovation (Modell, 2001). They frequently exhibit caution (Kane and Patapan, 2006), are risk averse (Hyson, 2013; Vigoda-Gadot et al., 2005), and make safe choices, thereby impeding innovation. This study argues that leaders in PSOs are frequently expected to excel at shaping ICSs. If leaders in PSOs are willing to change and innovate, they will generate a flow of creative ideas that will encourage the ICS to operate in novel ways while maintaining stability, thereby laying the groundwork for creativity and novel ways of working and improving performance in PSOs.

To address the research gap mentioned above, we constructed a moderator mediation model based on the resource-based view (RBV) (Barney, 1991), new public management (NPM) theory (Barzelay, 2019), and innovation systems theory (IST) (Schumpeter, 2017). In doing so, our study adds to the extant body of knowledge by clearly explaining an essential element for the ICS to promote positive innovation and the conversion of intensity of innovation into performance for PSOs, namely, transformational leadership. This study also makes a practical contribution by concluding that ICSs do not promote conservatism and stagnation in PSOs; conversely, when catalyzed by transformational leadership, they can promote innovation. Our empirical evidence of the interaction between transformational leadership and ICSs can serve as a guide for PSOs seeking to increase the intensity of innovation while maintaining stable operations to improve performance and sustainable development.

The remainder of our paper is structured as follows. The following section discusses the development of a theoretical model based on the underlying theories, namely, RBV, NPM, and IST, with particular emphasis on the relationships between ICS, intensity of innovation, and organizational performance, as well as the moderating effect of transformational leadership. Subsequently, a description of the research methodology and key findings is presented. The study's theoretical and practical implications are then discussed. The concluding section discusses the study's limitations and makes recommendations for future research.

## 2 Model and hypotheses development

### 2.1 The mediating role of intensity of innovation in PSOs

Given the critical role of public sector innovation in addressing the economic and social challenges confronting public sectors (Bloch and Bugge, 2013), an effective ICS will assist PSOs in optimizing their operations and utilizing resources efficiently. In addition to the rigor of the innovation plan, internal control is an element that ranks second in importance for innovation in PSOs (Freeman and Engel, 2007). Internal control will assist these organizations to establish appropriate goals and motivate employees to participate in the innovation process, exploit knowledge, and complete tasks, all of which will enable PSOs to focus their resources on innovation (Shen et al., 2020).

This study uses the RBV (Barney, 1991) to explain the effect of ICSs on the intensity of innovation by arguing that the ICS can satisfy the VRIN conditions. In this regard, the ICS is capable of achieving the VRIN conditions in the following ways: valuable – as demonstrated by PSOs refining processes and minimizing operational weaknesses; rare – the information and outputs of ICSs are specific in the public sector; inimitable – the design of the ICS depends on the specific characteristics and management needs of each PSO, and therefore it is difficult to imitate; and non-substitutable – ICSs are irreplaceable since maintaining ICSs is probably the most efficient way for PSOs to accomplish their established goals. Through arguments based on the RBV, it is demonstrated that an ICS which satisfies VRIN conditions is a valuable resource for assisting PSOs in achieving operational stability and establishing a foundation for innovation. This is due to the fact that an ICS assists organizations to improve compliance with laws and regulations, as well as mitigate unexpected risks associated with innovation activities (Li et al., 2006). Therefore, not only in Vietnam but throughout the public sector, organizations must place greater emphasis on internal control to maintain operational stability while also maximizing resource utilization and increasing intensity of innovation. Consequently, we propose the following hypothesis:

H1 ICS has a positive effect on the intensity of innovation.

According to Demircioglu and Audretsch (2017), the theoretical foundation system adequately reflects the prevalence of private sector innovation activities. However, in light of the public sector's need for innovation, researchers have not fully addressed this issue (Demircioglu and Audretsch, 2017). Moussa et al. (2018) assert, in particular, that intense global competition and rapid technological development have compelled PSOs to increase their intensity of innovation to successfully implement ideas and processes that address existing problems and create new opportunities. Additionally, a distinguishing feature of PSOs is their continued reliance on traditional modes of operation, as well as the bureaucratic issue that has harmed and stifled their innovation (Hjelmar, 2021; Parker and Bradley, 2000).

On the other hand, PSOs that adopt innovations create the most opportunities for innovation within their organizations, resulting in increased organizational performance (Shanker et al., 2017). When the NPM represents an evolution and renewal of historical trends in public administration, these innovations have built incrementally on previous modifications (Page, 2005). Indeed, the NPM is a framework that places a premium on evaluating the operating efficiency of PSOs (Osborne and Gaebler, 1992). According to this framework, clear incentives could aid public sector leaders in removing innovation barriers to improving organizational performance. As a result, under the pressure of the NPM, PSOs will make a concerted effort to develop solutions to regionally prevalent problems while also promoting the highest level of innovation. A high level of innovation will serve as a positive signal to encourage PSOs to reduce stagnation, increase performance, and establish a solid foundation for sustainable development (Potts and Kastelle, 2010). As a result, we propose the following hypothesis:

H2 Intensity of innovation has a positive effect on organizational performance.

Our study also proposes that ICS can indirectly affect organizational performance. An effective and efficient ICS can assist PSOs in combating fraud (Peltier-Rivest, 2018; Shonhadji and Maulidi, 2020), reducing bribery and corruption (Islam, 2014; Manurung et al., 2015), ensuring optimal resource utilization, increasing the efficiency of public assets, increasing transparency, and enhancing accountability (Aziz et al., 2015; Reginato et al., 2016). The above arguments suggest the mediating effect of intensity of innovation in the link between ICS and organizational performance. Accordingly:

H3 ICS positively affects organizational performance via the mediating role of intensity of innovation.

### 2.2 The moderating roles of transformational leadership in PSOs

The success of innovation practices in PSOs is contingent upon a variety of factors. These include not only institutional constraints (Van Waarden, 2001) but also technological and resource constraints (Bekkers, 2011; Boymal et al., 2007; Clausen et al., 2020), as well as the lack of leadership focus on innovation (Parry and Proctor-Thomson, 2002). Therefore, we argue that there are numerous opportunities for ICSs to be effective and efficient in PSOs with transformational leaders. To meet the control requirements necessary to facilitate innovation, PSOs will require more than transformational leaders to address the typical weaknesses of traditional leaders and thus increase the intensity of innovation. As a result, we introduce transformational leadership as a moderating variable in the relationship between ICSs and intensity of innovation. Transformational leaders are well known for elevating followers' aspirations for achievement while also promoting organizational development (Bass and Avolio, 1990). Bass and Avolio (1990) argue that transformational leaders will boost followers' confidence and shift their focus away from survival concerns and toward achievement, growth, and development. Transformational leaders are frequently viewed as visionaries and catalysts of organizational change in the public sector (Denhardt and Campbell, 2006).

According to IST, while transformational leaders recognize the critical role of ICSs in the innovation process, they seek to leverage the ICS effectively to promote innovation by developing strategies for innovation that are compatible with the ICS's actors, namely, followers. For instance, they frequently demonstrate their commitment to innovation by implementing appropriate internal control strategies, which include paying close attention to the internal control structure and procedures, addressing internal control deficiencies, enhancing knowledge mining, and other similar endeavors (Le et al., 2020; Shen et al., 2020).

We argue that when transformational leadership is more prevalent, the positive influence of the ICS on the intensity of innovation in PSOs will be increased. First, when transformational leadership is present, internal control can contribute to the intensity of innovation by directing resources from the ICS toward innovation activities. Due to the pressures on the public sector to provide new public services with diminishing resources (Clausen et al., 2020), internal control is a critical resource that assists organizations in safeguarding assets (Park et al., 2017) and effectively mitigating innovation risks. These factors have encouraged PSOs to work within the permissible boundary with confidence, as the ICS has already established a secure framework. Under the condition that the ICS exerts effective control over the organization's activities (Park et al., 2017), PSOs will feel secure in experimenting with new ideas, resulting in increased intensity of innovation. Second, the erroneous recognition of the unpopularity and ineffectiveness of transformational leadership was rejected when it was demonstrated that transformational leadership behaviors were found to be common and effective in PSOs (Dumdum et al., 2013). This indicates that transformation leadership will make every effort to effectively fulfill its critical role by establishing an ICS compatible with other foundational resources to positively affect intensity of innovation. Third, since transformational leaders positively affect employee satisfaction, beliefs, and behaviors (Bass et al., 1987), there is a tendency to focus on internal control mechanisms to increase intensity of innovation. As a result, employees will readily support leadership transformation. Therefore, the more committed and determined transformational leadership is in internal control activities, the greater the intensity of innovation. Additionally, transformation leaders will motivate employees and educate them on the importance of having an ICS in place to maximize the intensity of innovation (Jaskyte, 2012). Therefore, it is worth noting that PSOs with a higher level of transformation leadership cannot afford to overlook the responsibility of the leader to increase the intensity of innovation by strengthening the ICS. Based on the previous arguments driven by IST, we hypothesize that:

H4 Transformational leadership positively moderates the relationship between ICS and intensity of innovation.

**Figure 1:**
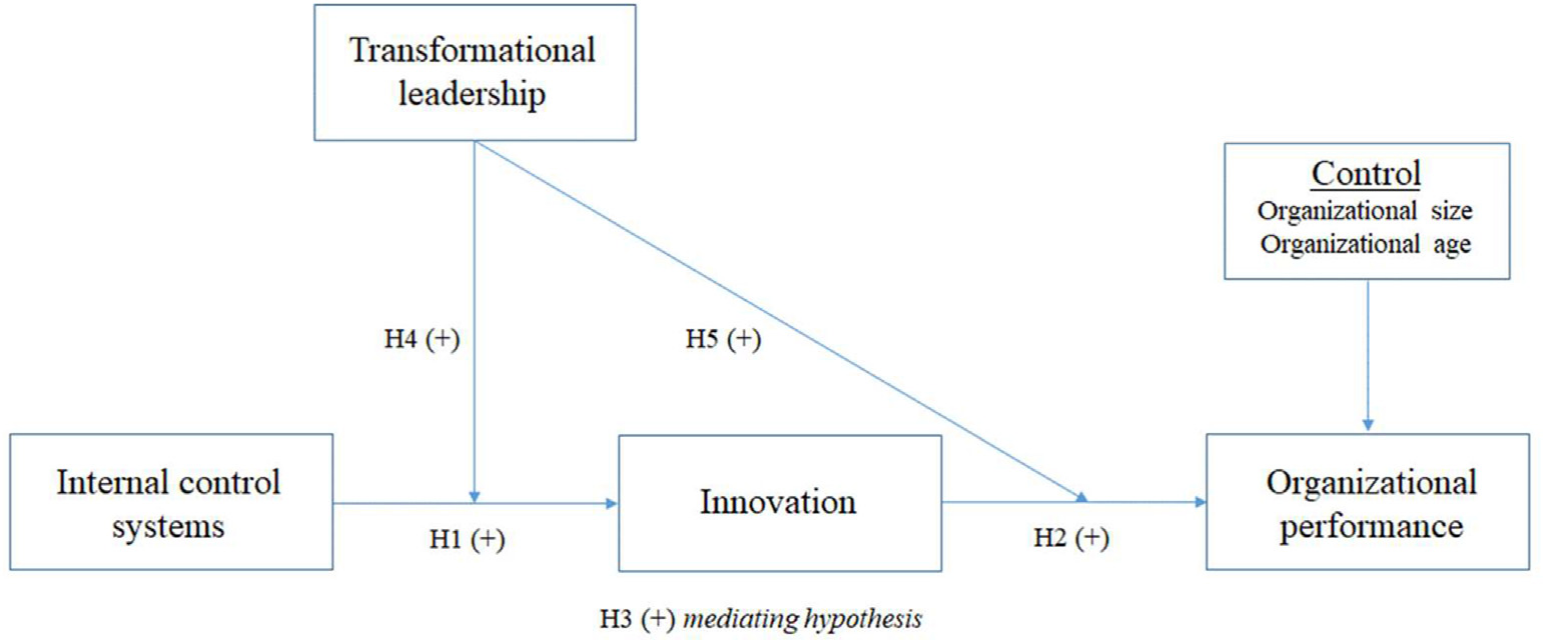
Proposed model and hypotheses.

PSOs have been confronted with new reform requirements (Stewart-Weeks and Kastelle, 2015) in which the government is required to respond not only to changes in the global environment but also to proactively and positively respond to the needs of the general public. The NPM trend has compelled PSOs to incorporate market mechanisms and business management principles into their operations and place a premium on customer satisfaction, thereby reforming and improving performance (Osborne and Gaebler, 1992). In a context where the public sector economy plays a secondary role but innovation is required (Bloch and Bugge, 2013), transformational leaders must play an active role in motivating employees through values and visions, future orientation, speech, and action to increase the intensity of innovation and performance. Specifically, under the influence of IST, transformational leaders will place a higher premium on innovation and work to convert its benefits into positive organizational outcomes (see Figure 1).

IST emphasizes that innovation does not occur in a vacuum but is dependent on the interaction of numerous different types of actors who participate and play various roles in the innovation process (Bloch and Bugge, 2013). Through the lens of IST, a transformational leader can be viewed as an ideal representative who is expected to influence internal actors, namely, followers, to accomplish organizational goals and increase effectiveness. We argue that, as critical drivers of the innovation process in PSOs, transformational leaders can engage in active dialogue with followers, leveraging their influence to accelerate the pace of change toward performance enhancement. In other words, interaction between transformational leaders and followers engaged in the innovation process in PSOs has the potential to synergize the innovative efforts of all organizational members, thereby increasing the impact of intensity of innovation on organizational performance. As a result, we propose the following:

H5 Transformational leadership positively moderates the relationship between intensity of innovation and organizational performance*.*

## 3 Methods

### 3.1 Research site

This study addresses the issues mentioned above in the context of the public sector in Vietnam, an emerging market where innovation is vital for performance management (Vu et al., 2021) but has not yet yielded positive results. While public sector innovation has received increased attention in Vietnam in recent years (Nguyen et al., 2021), the innovation process in the country continues to face numerous obstacles, including constraints on technological innovation (Boymal et al., 2007), resource constraints, risk aversion, passivity, and overreliance on public sector solutions to common problems (Kasabov, 2015). In addition, while ICSs in Vietnamese PSOs are not well-developed like those in developed countries, the implement internal control in Vietnamese PSOs is still in accordance with the COSO (2013) framework and primarily with the internal control guidelines specified in Vietnamese Standard Auditing No. 315 – VSA 315 in Circular 214/2012/TT-BTC (Ministry of Finance, 2012). Moreover, the Department of Legal Affairs and Internal Control was established in 2019 to assist internal control in reducing bribery, combating corruption, enhancing accountability, and enhancing organizational performance in Vietnam's public sector. Therefore, Vietnam is an appropriate case study for this research since it shares many issues with other transitional and developing countries, including low employee performance, systematic corruption, inefficiency, red tape, and power abuse (Pham, 2018). Furthermore, the public services in Vietnam had been significantly impacted by decades of bureaucratic and central planning, which is characterized by administrative orders and the "beg and give" mechanism (Painter, 2003).

Moreover, Vietnam was historically regarded as a socialist country that had endured protracted wars and always prevailed. Those wars led to Vietnam's economy being in a state of instability, fragility, and backwardness (Tran, 2018). Simultaneously, the efforts of the nation to boost the intensity of innovation to achieve political stability and refocus on economic recovery have been significantly harmed by the state bureaucracy, power, and central planning mechanism of bureaucracy and subsidies (Painter, 2005). As a result, Vietnam was classified as one of the poorest countries with per capita GDP of US$203 in 1986 (Voeten and Naude, 2014). However, since the 1980s, the Vietnamese Communist Party has implemented a program called "Doi Moi" (Renovation) that promotes economic, cultural, social, and political innovation. This historic turning point has led to the infrastructure being overhauled through a comprehensive market-oriented reform program (Thu et al., 2018). Since then, the bureaucracy issue has been somewhat alleviated, allowing the renovation process to proceed more smoothly.

Finally, Vietnam has a culture that values both power distance and leadership-focused power (Nguyen et al., 2019; Tran et al., 2017), and the majority of leaders are still heavily influenced by a collectivist culture that appears to be ingrained in their subconscious (Tran et al., 2017). Therefore, transformational leadership is required in Vietnam's PSOs to improve traditional ways of working and to focus on developing ICSs that maximize the use of common assets, thereby positively impacting the intensity of innovation (Jaskyte, 2012). In this context, transformational leaders in Vietnam's PSOs are expected to pay increased attention to the role and benefits of internal control, as the benefits of internal control for the public sector, such as increased efficiency in use of public assets, increased transparency in reporting, and increased compliance (Park et al., 2017; Reginato et al., 2016), as well as fostering innovation, are already evident in developed economies (Freeman and Engel, 2007). However, while innovation is critical for PSOs' performance in Vietnam (Vu et al., 2021), internal control in the public sector has received little attention. Therefore, Vietnam is an appropriate setting for studying PSOs and issues related to internal control and performance improvement through innovation.

### 3.2 Sampling and data collection

We approached PSOs in Vietnam with an independent internal control department or under internal control supervision of their agencies. The target respondents in this study are middle-level managers employed by Vietnamese PSOs who have a minimum of three years of experience in their respective represented organizations. These conditions are in place to ensure that respondents have the necessary expertise and knowledge to complete the questionnaire on behalf of their organizations. In addition, the mid-level managers are responsible for processing a large amount of data at multiple levels (from operational to strategic) (Mahama and Cheng, 2013), and therefore they are able to complete the questionnaire on behalf of an organization. Finally, rather than having leaders (senior managers) self-evaluate, mid-level managers can evaluate their leaders, usually top managers, by answering survey questions about transformational leadership.

The questionnaire was developed based on the scale items of the main constructs. It was written in English and then translated into Vietnamese by bilingual researchers following the back-translation procedure recommended by Brislin (1970). Subsequently, the questionnaire was reverse translated from Vietnamese into English to eliminate any translation errors. We distributed surveys via email and collected data from an independent research company. Because data collection from public sector organizations in Vietnam is rather difficult, a convenient sampling approach was used. To mitigate common method bias (Podsakoff et al., 2003), we collected data in two phases. In phase one, data on ICS and transformational leadership, as well as demographic information were collected. The intensity of innovation and organizational performance were assessed in phase two. Between the two phases, a short period of one month was chosen to minimize the dropout rate and memory bias (Einarsen et al., 2009). The two phases of data collection were matched using the unique identifier allocated to each respondent. Finally, given the organizational level of analysis, we searched the sample thoroughly to find multiple responses from the same organization. No such cases were identified during the scanning procedure. The demographic information of the respondents and their organizations is shown in Table 1.

**Table 1:** Demographic information (n = 319).

	n	%		n	%
*Work experience*			*Organizational age*		
3–5 years	13	4.1	<5 years	6	1.9
6–10 years	94	29.4	5–10 years	23	7.2
10–20 years	177	55.5	11–20 years	98	30.7
Over 20 years	35	11.0	21–50 years	167	52.4
*Organizational size (employees)*			<50 years	25	7.8
<50	23	7.2	*Organizational type*		
51–200	132	41.4	State enterprises	21	6.6
201–500	145	45.5	Administrative agencies	75	23.5
501–1,000	16	5.0	Public service units	143	44.8
>1,000	3	0.9	Other socio-political organizations	80	25.1

### 3.3 Measurement scales

We used well-established scales in the literature to measure the main constructs in the proposed model. Specifically, ICS was measured using the formative scale proposed by Chiu and Wang (2019), which was developed based on the COSO framework. The scale for intensity of innovation was based on the Innobarometer 2010 survey, which was inspired by the Oslo Manual (OECD, 2005) but adapted to the public administration context (Arundel et al., 2015) and used in subsequent studies, such as the study conducted by Clausen et al. (2020). To measure organizational performance, we adopted the seven-item scale developed by Speklé and Verbeeten (2014) and Verbeeten and Speklé (2015). In line with previous studies (Campbell, 2018; Wright et al., 2012), the moderator variable, namely, transformational leadership, was measured by a five-item scale popular in the public administration literature. Finally, we employed control variables for the performance of public organizations, including the organizational size (Gomes et al., 2017) and organizational age (Glisson and Martin, 1980). Table 2 shows the scale items of all the main variables in the proposed model.

**Table 2:** Scale evaluation.

Item	Weight/loading	*t*-value
**Internal control systems**		
*Control environment (formative scale)∗*		
• The organization demonstrates a commitment to integrity and ethical values.	0.39	2.87
• The board of directors demonstrates independence from management and exercises oversight of the development and performance of internal control.	(0.02)	0.18
• Management establishes, with board oversight, structures reporting lines, and appropriate authorities and responsibilities: in the pursuit of objectives.	0.18	1.30
• The organization demonstrates a commitment to attract, develop, and retain competent individuals in alignment with objectives.	0.23	1.28
• The organization holds individuals accountable for their internal control responsibilities in the pursuit of objectives	0.39	2.87
*Risk assessment (formative scale)∗*		
• The organization specifies objectives with sufficient clarity to enable the identification and assessment of risks relating to objectives.	0.43	2.53
• The organization identifies risks to the achievement of its objectives across the entity and analyzes risks as a basis for determining how the risks should be managed.	0.49	2.33
• The organization considers the potential for fraud in assessing risks to the achievement of objectives.	0.18	1.17
• The organization identifies and assesses changes that could significantly impact the system of internal control.		
*Control activities (formative scale)∗*		
• The organization selects and develops control activities that contribute to the mitigation of risks to the achievement of objectives to acceptable levels	0.39	1.87
• The organization selects and develops general control activities over technology to support the achievement of objectives.	0.25	1.14
• The organization deploys control activities through policies that establish what is expected and in procedures that put policies into action.	0.52	2.69
*Information and communication (formative scale)∗*		
• The organization obtains or generates and uses relevant, quality information to support the functioning of internal control.	0.13	0.70
• The organization internally communicates information, including objectives and responsibilities for internal control, necessary to support the functioning of internal control.	0.60	2.42
• The organization communicates with external parties about matters affecting the functioning of internal control.	0.40	1.43
*Monitoring (formative scale)∗*		
• The organization selects, develops, and performs ongoing and/or separate evaluations to ascertain whether the components of internal control are present and functioning.	0.63	2.47
• The organization evaluates and communicates internal control deficiencies in a timely manner to those parties responsible for taking corrective action, including senior management and the board of directors, as appropriate.	0.45	1.44
**Transformational leadership CR = 0.91; AVE = 0.69)**		
• My leader clearly articulates his/her vision of the future	0.85	29.94
• My leader leads by setting a good example.	0.86	34.48
• My leader challenges me to think about old problems in new ways.	0.86	34.45
• My leader says things that make employees proud to be part of the organization.	0.70	12.44
• My leader has a clear sense of where our organization should be in five years.	0.89	56.44
**Intensity of innovation (summated scale)**		
*Since last year, has your organization introduced any new or significantly improved (1 = Yes. 0 = No)*		
• services?	-	-
• methods of communicating your activities to the public, such as new or improved methods of promoting your organization or your services?	-	-
• methods of communicating your activities to the public, such as new or improved methods of influencing the behavior of users, citizens or others?	-	-
• processes or organizational methods, such as new or improved methods of providing services or interacting with your users?	-	-
• processes or organizational methods, such as new or improved delivery or logistics systems for your inputs?	-	-
• processes or organizational methods, such as new or improved supporting activities such as maintenance systems, purchasing, accounting, or computing systems, etc.?	-	-
• processes or organizational methods, such as new or improved management systems?	-	-
• processes or organizational methods, such as new or improved methods of organizing work responsibilities or decision-making?	-	-
**Organizational performance CR = 0.92; AVE = 0.63)**		
• The quantity or amount of work produced	0.76	37.03
• The quality or accuracy of work produced	0.78	39.55
• The number of innovations or new ideas by the unit	0.80	42.54
• Reputation of ‘‘work excellence”	0.79	44.28
• Attainment of unit production or service goals	0.81	46.10
• Efficiency of unit operations	0.81	41.69
• Morale of unit personnel	0.81	49.08

Note: ∗: CR and AVE are not applicable for formative and summate scales.

### 3.4 Common method bias and multicollinearity issues

As we collected data from key informants, common method bias may be a concern, resulting in measurement error and jeopardizing the validity of conclusions about the dimensions' interrelationships (Podsakoff et al., 2003). First, we performed the Harman single factor test to rule out any possible common method bias. The findings suggested that no single factor accounted for the significant share of the variance (the first factor accounted for 32.75% of the 64.27% explained variance). Second, we used the marker-variable technique since the Harman test is highly conservative in detecting bias (Lindell and Whitney, 2001; Malhotra et al., 2006). The questionnaire item "Do you like swimming?" was employed as a marker variable since it has no theoretical association with any of the variables in this study. After rM effects were removed, the mean change in the correlations between the primary constructs (rU–rA) was only 0.03. Therefore, all the tests listed above indicate that this study is free of common method bias. Moreover, we investigated the possibility of multicollinearity. The maximum inflation value for the inner variance was 3.71, much less than the high threshold value of 10 (O'Brien, 2007). Consequently, the level of multicollinearity in this study is insignificant.

## 4 Results

### 4.1 Reliability and validity tests

To estimate the measurement and structural models, we used Partial Least Squares Structural Equation Modeling (PLS-SEM) with SmartPLS v.3.3.3. The reliability and validity of the main constructs are demonstrated in Table 2 utilizing composite reliability (CR), average variance extracted (AVE), and the outer weights and loadings of the scale items, as well as their related t-values. The outer loadings for two reflective constructs (i.e., transformational leadership and organizational performance) ranged between 0.70 and 0.89 and are therefore not below the 0.70 cut-off value. Moreover, all these reflective constructs have AVE values greater than 0.50. These results imply that the measurement model has an adequate level of convergent validity. Furthermore, the CR values for the reflective scales were all above 0.70, indicating that the measurement scales are highly reliable (Kline, 2016).

The discriminant validity analysis is illustrated in Table 3. The absolute values of the correlation coefficients between any pair of constructs (which ranged from 0.00 to 0.80) were consistently less than their square root of AVE values (which ranged from 0.83 to 1.00) and did not exceed their CR values. These findings imply that the discriminant validity is excellent (Fornell and Larcker, 1981). In addition to the approach utilized by Fornell and Larcker (1981), we evaluated discriminant validity using a more rigorous Heterotrait–Monotrait (HTMT) test (Henseler et al., 2015). The calculated bootstrapped HTMT values between 0.17 and 0.60 were significantly less than the harsher criteria cut-off value of 0.85 (Henseler et al., 2015), providing more solid evidence of discriminant validity.

**Table 3:** Discriminant validity analysis.

	1	2	3	4	5	6	7	8
1. Control environment	N/A							
2. Risk assessment	0.80∗∗	N/A						
3. Control activities	0.70∗∗	0.76∗∗	N/A					
4. Information and communication	0.73∗∗	0.74∗∗	0.73∗∗	N/A				
5. Monitoring	0.72∗∗	0.67∗∗	0.69∗∗	0.78∗∗	N/A			
6. Transformational leadership	(0.01)	0.00	0.04	0.02	0.07	**0.83**		
7. Intensity of innovation	0.19∗∗	0.20∗∗	0.21∗∗	0.22∗∗	0.22∗∗	0.17∗∗	**1.00**	
8. Organizational performance	0.14∗	0.13∗	0.18∗∗	0.16∗∗	0.19∗∗	0.19∗∗	0.57∗∗	**0.80**

Notes: correlation between variables (off diagonal); square root of AVE (bold diagonal); ∗,∗∗ – correlation is significant at the 5% and 1% levels, respectively (2-tailed t-test); N/A – square root of AVE is not applicable for formative constructs.

### 4.2 Results of hypotheses testing

We used PLS-SEM to analyze the proposed model and hypotheses. The sample size of 319 was appropriate since it surpasses tenfold the maximum number of possible paths to any construct in the model (Hair et al., 2017). Additionally, the proposed model has a standardized root mean squared residual of 0.089, which is less than the 0.09 threshold established by Hu and Bentler (1999), indicating that the proposed model fits the data sufficiently.

To test the hypotheses, we created three hierarchical models (see Table 4). Model 1 illustrates the direct relationship between ICS and organizational performance. Model 2 is identical to Model 1 but includes intensity of innovation as a mediator. Model 3 adds transformational leadership as the moderator of the relationships between ICS and intensity of innovation, and between intensity of innovation and organizational performance.

**Table 4:** Results of hypotheses testing.

	Model 1	Model 2		Model 3	
	PERF	INNO	PERF	INNO	PERF
*Independent variable*					
ICS	0.22	0.24	0.08	0.25	0.03
	(3.90)^c^	(4.13)^c^	(1.45)	(4.41)^c^	(0.49)
TL				0.17	0.11
				(3.13)^c^	(2.58)^b^
INNO			0.56		0.54
			(13.80)^c^		(13.68)^c^
TL × ICS				0.21	
				(3.77)^c^	
TL × INNO					0.22
					(5.59)^c^
*Control variable*					
Organizational size	0.06		0.09		0.09
	(1.03)		(2.19)^b^		(2.02)^b^
Organizational age	0.08		0.06		0.06
	(1.43)		(1.30)		(1.30)
Adjusted R^2^ of PERF	0.05		0.34		0.40
Mediating effect			Estimate	LLCI	ULCI
ICS→INNO→PERF			0.14	0.08	0.21
			(3.90)^c^		

Notes: ICS – internal control system; INNO – intensity of innovation; TL – transformational leadership; PERF – organizational performance; TL × ICS – interaction between TL and ICS; TL × INNO – interaction between TL and INNO; a, b, c – significance at the 10%, 5%, and 1% levels, respectively (2-tailed t-test); LLCI – lower level of confidence interval; ULCI – upper level of confidence interval.

H1 asserted that ICS has a beneficial effect on intensity of innovation in PSOs, which is corroborated by our analysis (model 1: β = 0.22; t-value = 4.90). Additionally, our analysis supports H2, which hypothesized that intensity of innovation improves organizational performance (model 2: β = 0.56; t-value = 13.80; model 3: β = 0.54; t-value = 13.68). In addition, the indirect effect of ICS on organizational performance via intensity of innovation was significant (β = 0.14; t-value = 3.90; 95% CI = 0.08; 0.21), indicating that H3 regarding the mediating effect of intensity of innovation on the relationship between ICSs and organizational performance is supported. Moreover, when intensity of innovation was introduced as the mediator, the effect of ICS on organizational performance changed from significant (model 1: β = 0.22; t-value = 3.90) to insignificant (model 2: β = 0.08; t-value = 1.45), suggesting that intensity of innovation fully mediates the relationship between ICS and organizational performance. Thus, H3 is confirmed.

To examine H4 and H5 regarding the positive moderating effects of transformational leadership on the relationships between ICS and intensity of innovation and between intensity of innovation and organizational performance, we created two interaction terms, TL × ICS and TL × INNO, after mean centering the independent variable (i.e., ICS and intensity of innovation) and the moderating variable (i.e., transformational leadership) on avoiding multicollinearity (Aiken et al., 1991). The two interaction terms had a positive and significant effect on intensity of innovation (model 3: β = 0.21; t-value = 3.77) and organizational performance (model 3: β = 0.22; t-value = 5.59), thus supporting H4 and H5.

**Figure 2:**
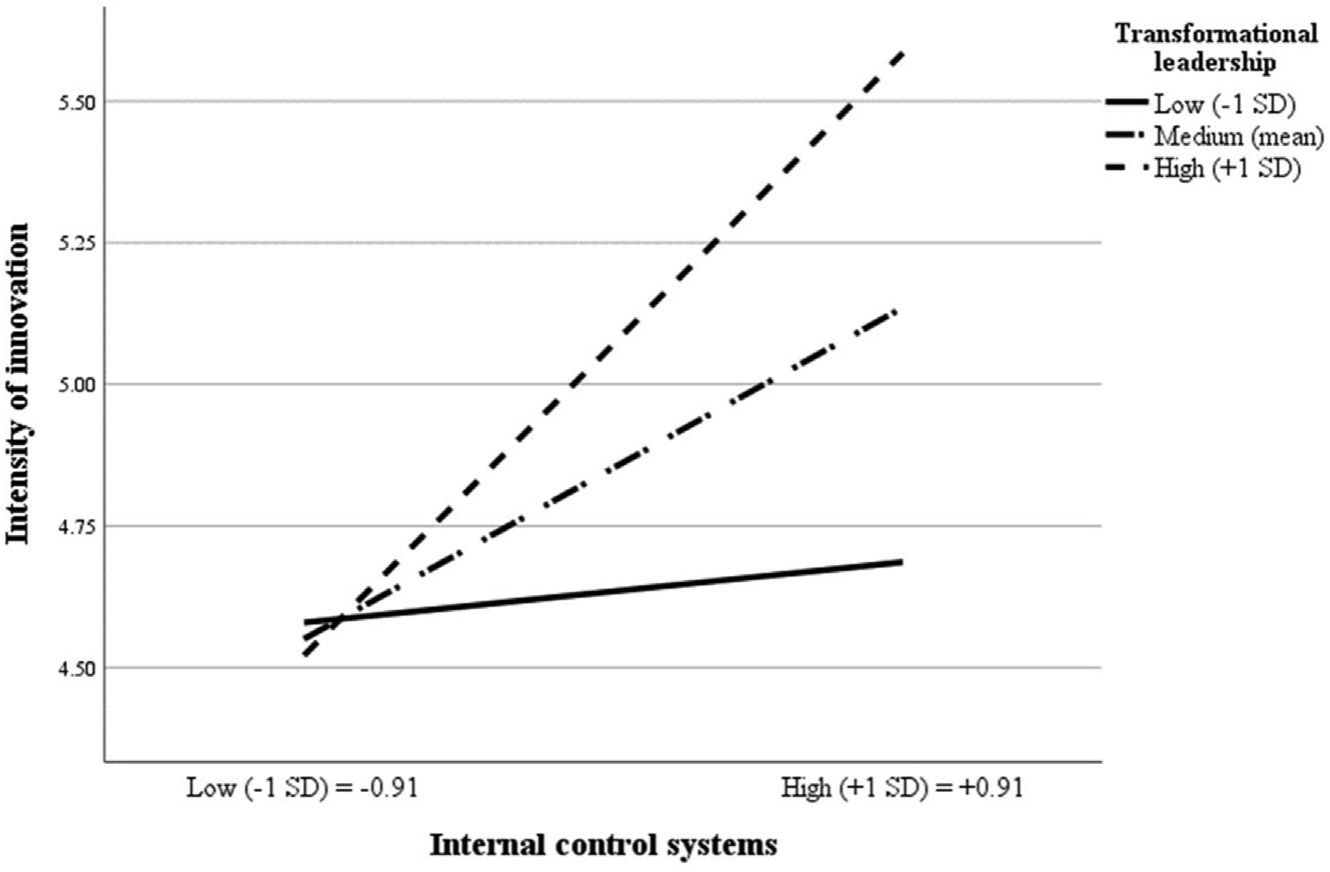
Interaction effect of ICSs with transformational leadership on intensity of innovation.

**Figure 3:**
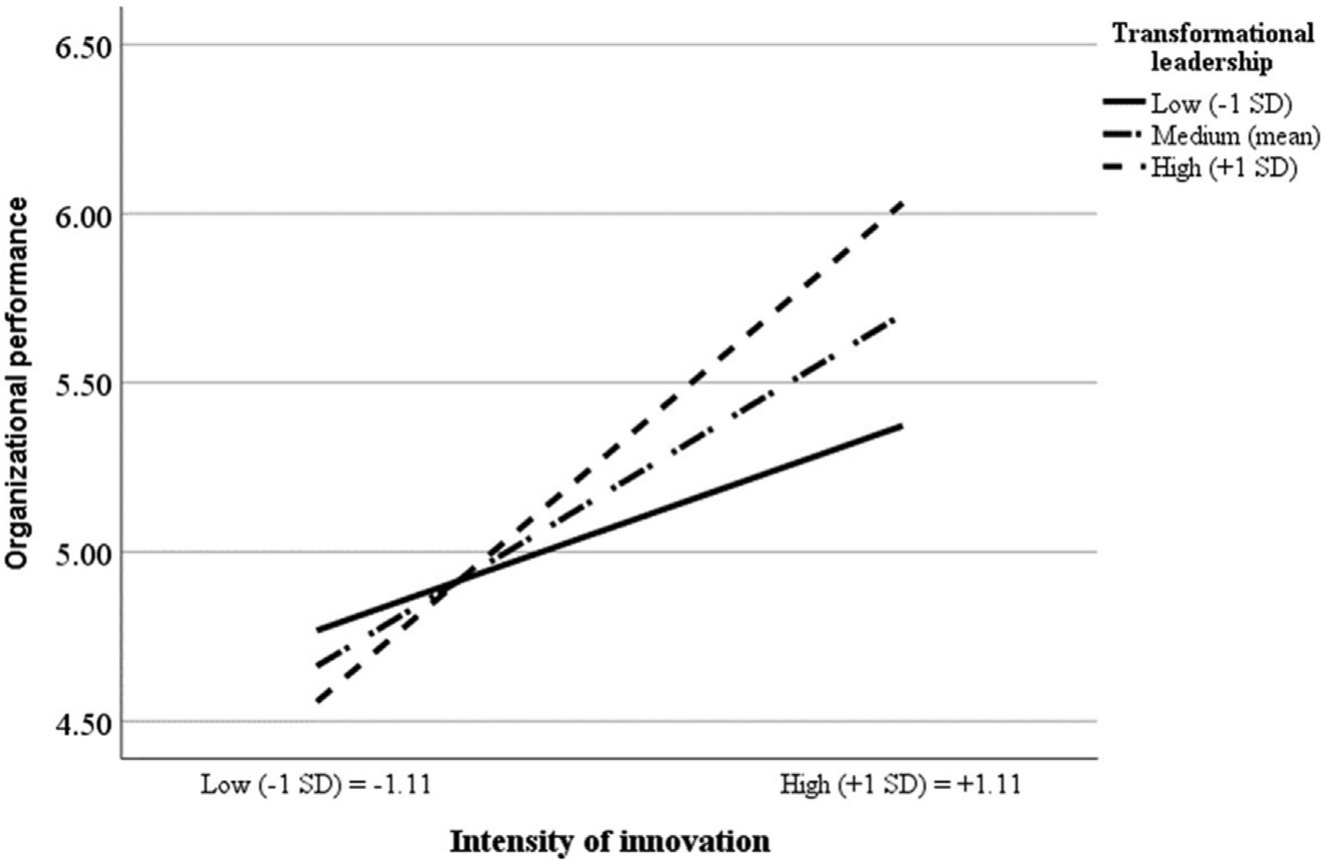
Interaction effect of intensity of innovation with transformational leadership on organizational performance.

To better understand the nature of these interactions, we followed Aiken et al. (1991) to plot the effect of internal control structure on intensity of innovation and the impact of intensity of innovation on organizational performance at low (−1 SD) and high (+1 SD) levels of transformational leadership. The interaction graphs (Figures 2 and 3) demonstrate that when transformational leadership is low (high), these effects are low (high). These results suggest that ICS and innovation tend to be less (more) effective in PSOs with a low (high) level of transformation leadership. This finding supports H4 and H5.

### 4.3 Discussion

This section discusses the hypothesis testing results. First, the acceptance of H1 indicates that effective ICSs enable PSOs to set appropriate goals, motivate employees to participate in the innovation process, exploit knowledge, and complete tasks, all of which allow PSOs to focus their resources on innovation (Shen et al., 2020), thereby increasing the intensity of innovation. Moreover, the confirmation of H1 supports the RBV (Barney, 1991) in the internal control context and aligns with previous accounting studies in Vietnam (e.g., Nguyen, 2018; Tu and Nguyen, 2021). This finding also suggests that RBV is a relevant theory for explaining the relationship between accounting resources (e.g., management accounting systems, ICSs) and competitive advantage.

Second, confirmation of H2 lends support to the NPM theory (Barzelay, 2019) in the context of innovation research in Vietnam, where innovation is critical for the performance of PSOs in the country (Vu et al., 2021). It demonstrates that NPM is an appropriate framework for explaining the relationship between innovation intensity and performance in Vietnamese PSOs.

Third, the support of H3 indicates that intensity of innovation is a necessary mediator connecting ICSs to enhanced PSO performance. This is a novel finding in comparison to previous studies conducted exclusively in private organizations focusing exclusively on the direct impact of ICSs on risk management, corporate governance, and financial efficiency (e.g., Al-Thuneibat et al., 2015; Khlif et al., 2019), or on the direct effect of ICS on organizational performance outcomes (e.g., Tetteh et al., 2020).

Fourth, while knowledge about the moderating effect of transformational leadership on the direct effect of ICS on the intensity of innovation remains limited in PSOs in emerging market contexts, the support of H4 for the moderating role of transformational leadership is novel. This finding implies that if leaders of PSOs are willing to transform in an emerging market context, such as Vietnam, the intensity of innovation can be increased through well-organized ICSs.

Finally, the test of H5 revealed that transformational leadership could leverage the effect of innovation intensity on PSO performance. This demonstrated that when PSOs are innovative and have a high degree of innovation, transformational leaders can use their influence to accelerate the pace of change toward performance enhancement. Thus the impact of innovation intensity on performance can be increased. By accepting H4 and H5, our study sheds light on the critical role of transformational leadership in the public sector context. Based on IST, it has been demonstrated that transformational leadership is effective and necessary in PSOs, while also catalyzing PSO innovation systems by promoting the innovative potential of ICSs.

## 5 Implications and future research directions

In response to the call for investigating public sector innovation (Clausen et al., 2020), our study investigated the mediation and moderation processes that connect ICSs to the level of innovation and organizational performance in PSOs in an emerging market. Drawing on the RBV (Barney, 1991), NPM theory (Barzelay, 2019), and IST (Schumpeter, 2017), we hypothesized that intensity of innovation mediates the relationship between ICSs and organizational performance in PSOs in an emerging country and that the degree of the mediating effects is contingent upon transformational leadership. Data from 319 PSOs in Vietnam support our proposed model and hypotheses. The findings have some theoretical and managerial implications, which we will discuss in turn.

### 5.1 Theoretical implications

Our study contributes to the existing literature in several ways. First, the positive effect of ICS on the intensity of innovation found in our study responds to the skepticism about the beneficial role of ICS in innovation. It can be demonstrated that, when faced with the NPM pressure, PSOs seek to leverage their ICS resources in accordance with VRIN conditions to increase the intensity of innovation and thus enhance organizational performance.

Second, we revealed the critical role that transformation leadership can play in increasing the intensity of innovation in PSOs to increase efficiency, effectiveness, and performance (Demircioglu and Audretsch, 2019). Specifically, we identified the vital role of transformational leadership in improving the intensity of innovation in PSOs to boost their organizational performance. This timely contribution reflects the growing emphasis on the moderating role of transformational leadership in the innovation process of PSOs to improve performance (Shanker et al., 2017; Sun and Henderson, 2017) via the establishment and operation of an effective ICS. The resulting insights demonstrate the critical role of transformational leadership in increasing the intensity of innovation of PSOs through the effective use of ICS resources for innovation activities.

Finally, our research sheds new light on the vital role of transformational leadership in the context of the public sector. While several previous studies (e.g., Bass and Riggio 2006; Shamir and Howell, 2018) suggest that transformational leadership is less prevalent and effective in PSOs than in private sector organizations, our research, grounded in IST, provides a counter view by proving that transformational leadership is effective while it can still act as a catalyst for the PSO innovation systems by promoting the innovative potential of ICSs.

### 5.2 Managerial implications

Our study has several managerial implications. First, the impact of ICSs on organizational performance emphasizes the importance of ICS implementation in PSOs in emerging countries and the role of the policymakers in fostering this process. While ICSs have been widely implemented in private enterprises in emerging markets such as Vietnam, they have not been widely implemented in the public sector or received much attention. Although internal control policies in Vietnam have been developed in accordance with international standards, they have not been fully updated in comparison to developed countries. As a result, we recommend that emerging economies such as Vietnam update their internal control laws and regulations to include specific guidelines for PSOs to contribute more efficiently to the management of ICSs. Furthermore, PSOs should place a higher premium on ICSs to foster innovation (Jaskyte, 2012) and boost performance to adapt to the pressure to provide new public services with scarce resources (Clausen et al., 2020).

Second, the findings of the full mediating role of intensity of innovation in the relationship between ICS and organizational performance indicate that PSOs must improve the conditions that foster the intensity of innovation to reap the benefits of ICSs. These conditions include proactive employee control over work, motivation to improve performance, and feedback to employees from leaders (Deci and Ryan, 2000; Demircioglu and Audretsch, 2017).

Finally, the crucial role of transformational leadership is evident in the transformation of intensity of innovation into superior performance in the public sector context. These findings are also consistent with IST, which asserts that innovation does not occur in isolation but requires interaction between ICSs and leaders, which are the actors involved in the process of innovation. Through the moderating role of transformational leadership in the ICS – innovation – performance chain, the synergies between these actors were highlighted in this study. This suggests that PSOs should maximize their use of transformational leadership and ICSs to increase their intensity of innovation and thus their performance outcomes (Shanker et al., 2017).

### 5.3 Limitations and future research directions

Despite these significant contributions, the current study has several noteworthy limitations. First, this study was cross-sectional, which limited inferences about causal relationships between ICS, intensity of innovation, and organizational performance. Consequently, a follow-up longitudinal study is required to confirm these relationships. Second, researchers should exercise caution when extrapolating our findings to PSOs in other countries. Additional research should be conducted in other emerging countries to examine the effects of unique contextual factors (e.g., culture, institutions, and politics) on the relationships in our study to obtain additional insights. Third, as obtaining objective data on organizational performance from PSOs in Vietnam is challenging due to privacy concerns, this study relied on self-reported data from managers. Therefore, future research should collect objective data to evaluate organizational performance. Finally, our study examined PSO performance using only two control variables, namely, organizational size and organizational age. Additional control variables (e.g., resource availability, financial autonomy) that may affect the performance of PSOs could be included in subsequent research.

## Declarations

### Author contribution statement

Tu Thanh Hoai: Conceived and designed the experiments; Analyzed and interpreted the data; Contributed reagents, materials, analysis tools or data; Wrote the paper.

Bui Quang Hung: Conceived and designed the experiments; Contributed reagents, materials, analysis tools or data; Wrote the paper.

Nguyen Phong Nguyen: Conceived and designed the experiments; Analyzed and interpreted the data; Wrote the paper.

### Funding statement

This work was supported by University of Economics Ho Chi Minh City (UEH) under Grant number 2021-12-01-0703.

### Data availability statement

Data will be made available on request.

### Declaration of interests statement

The authors declare no conflict of interest.

### Additional information

No additional information is available for this paper.
